# Lipids and Alzheimer’s Disease

**DOI:** 10.3390/ijms21041505

**Published:** 2020-02-22

**Authors:** Yu-Chia Kao, Pei-Chuan Ho, Yuan-Kun Tu, I-Ming Jou, Kuen-Jer Tsai

**Affiliations:** 1 Institute of Clinical Medicine, College of Medicine, National Cheng Kung University, Tainan 704, Taiwan; yukanomail2006@yahoo.com.tw (Y.-C.K.); peggy821124@gmail.com (P.-C.H.); 2 Department of Pediatrics, E-DA Hospital, Kaohsiung 824, Taiwan; 3 Department of Orthopedics, E-DA Hospital, Kaohsiung 824, Taiwan; ed100917@edah.org.tw (Y.-K.T.); jming@mail.ncku.edu.tw (I.-M.J.); 4 Research Center of Clinical Medicine, National Cheng Kung University Hospital, College of Medicine, National Cheng Kung University, Tainan 704, Taiwan

**Keywords:** Alzheimer disease, lipids, fats, fatty acids, cholesterols, lipidomics

## Abstract

Lipids, as the basic component of cell membranes, play an important role in human health as well as brain function. The brain is highly enriched in lipids, and disruption of lipid homeostasis is related to neurologic disorders as well as neurodegenerative diseases such as Alzheimer’s disease (AD). Aging is associated with changes in lipid composition. Alterations of fatty acids at the level of lipid rafts and cerebral lipid peroxidation were found in the early stage of AD. Genetic and environmental factors such as apolipoprotein and lipid transporter carrying status and dietary lipid content are associated with AD. Insight into the connection between lipids and AD is crucial to unraveling the metabolic aspects of this puzzling disease. Recent advances in lipid analytical methodology have led us to gain an in-depth understanding on lipids. As a result, lipidomics have becoming a hot topic of investigation in AD, in order to find biomarkers for disease prediction, diagnosis, and prevention, with the ultimate goal of discovering novel therapeutics.

## 1. Background

There were ~46.8 million dementia cases worldwide in 2015 according to the world Alzheimer report, with an estimated 9.9 million new cases per year [1], indicating the global burden of dementia. Alzheimer’s disease (AD), the most common type of dementia, is a neurodegenerative disease clinically characterized by progressive memory loss, cognitive dysfunction and behavioral change. The major neuropathological hallmark of AD is the accumulation of amyloid protein in senile plaques due to over-production or impaired clearance of β-amyloid (Aβ) peptides and the deposition of neurofibrillary tangles (NFTs), which give rise to synaptic loss and neurodegeneration.

Cerebral lipids account for at least 50% of dry brain weight—the most lipid-rich organ next to the adipose tissue [2]. Lipids are the basic structural component of neuronal cell membranes. Brain lipids are comprised of 50% phospholipids, below 40% glycolipids, 10% cholesterol, cholesterol ester and traces of triglycerides. Long-chain polyunsaturated fatty acids (LC-PUFAs) account for 25–30% of the total fatty acids (FAs) in the human brain, including docosahexaenoic acid (DHA) and arachidonic acid (AA) [2]. Cerebral lipid peroxidation was found to be an early event in AD. Brains of AD patients display a higher number of lipoid granules (or adipose inclusions) in glia, suggesting aberrant lipid metabolism. Genome-wide association studies (GWAS) have found associations between AD and several genes involved in lipid homeostasis such as *APOE* (apolipoprotein E), *CLU* (clusterin, also known as apolipoprotein J), *SORL1* (sortilin-related receptor 1) and *ABCA7* (ATP-binding cassette, sub-family A, member 7) [3].

Lipid analyses—such as liquid chromatography or gas chromatography-mass spectrometry and mass spectrometry imaging—enable the identification of lipids in tissues and body fluids [4,5]. There are eight categories of lipids, as listed in Table 1. In the following sections, we will discuss research regarding each lipid group and their connection with AD.

## 2. Lipid Rafts

The lipid compositions of the two membrane monolayers are different: the inner leaflet is enriched in phosphatidylserine, phosphatidylethanolamine and phosphatidylinositol, whereas the outer leaflet is enriched in phosphatidylcholine and sphingomyelin [6]. Lipid rafts are dynamic structures within the cell membranes that play crucial roles in signal transduction, cell adhesion and lipid/protein sorting. Lipid rafts are characterized by combinations of sphingolipids, cholesterol, saturated FAs and a reduced content of PUFAs. Many AD-associated proteins have been found in lipid rafts such as amyloid-β protein precursor (βAPP), β-secretase, γ-secretase and neprilysin [7]. Lipid rafts serve as platforms where Aβ interacts with ApoE, and tau to promote the aggregation of Aβ oligomers and hyperphosphorylated tau [8]. Aβ production (amyloidogenesis) is related to lipid composition within lipid rafts [9]. The formation of cytotoxic Aβ fibrils is triggered by lipid rafts including GM1 ganglioside, cholesterol and sphingomyelin [10]. Lipid rafts from frontal cortex of AD patients exhibit abnormally low contents of n-3 LC-PUFAs (mainly DHA) and monoenes (mainly oleic acid), as well as significant reductions in the unsaturation and peroxidability indexes compared to healthy control [11]. These FA alterations in the level of lipid rafts were observed early in AD pathogenesis, particularly in the frontal and entorhinal cortices [12].

## 3. Aging and Lipids

During the first two decades of human life, amounts of cerebral lipids increase and then begin to gradually decrease after the age of 50 [13]. Aging causes alterations of adipose tissue distribution with an elevation of systemic free fatty acids (FFA) levels, a common feature of metabolic syndromes [14]. There are age-related alterations of lipid compositions in different brain areas. The saturated fatty acids (SFA), monounsaturated fatty acids (MUFA) and PUFA are significantly greater in mid-life males compared to younger males, whereas PUFAs including DHA, AA decrease and MUFAs increase in the grey matter of orbitofrontal cortex with aging [15]. Likewise, aging is related to increased inflammation. Lipids are the mediators that orchestrate many immune responses. Some specialized pro-resolving lipid mediators (SPMs) are particularly associated with aging [16].

## 4. Blood–Brain-Barrier (BBB)

There is a negative correlation between cerebral blood flow (CBF) and age [17]; moreover, blood–brain barrier (BBB) permeability is greater in older compared to younger individuals [18]. Dynamic contrast magnetic resonance imaging (MRI) suggests that BBB breakdown is an early event that begins in the hippocampus of the aging brain [19]. Brain hypoperfusion and loss of BBB integrity result in reduced energy availability and disrupted synapses, leading to impairments in memory and learning [20]. BBB integrity is compromised in AD [21] as evidenced by the detection of plasma proteins in the parenchyma of AD brains as a consequence of cerebrovascular changes (breach) called cerebral amyloid angiopathy. In total gray matter and cortex of the brain, the BBB leakage was significantly increased in AD patients compared with in control subjects and correlated with disease severity [22].

Takechi et al. showed that after 12 weeks of high-fat diet (HFD), the BBB dysfunction of SFA-diet mice was 30-fold greater than the control group while that on high-cholesterol diet was 7-fold increased. When these mice were given probucol, which is the lipid-lowering agent, the non-esterified FA levels of plasma were increased and cerebrovascular inflammation or loss of BBB integrity didn’t occur, suggesting that probucol prevents disturbances of BBB induced by HFDs by suppressing inflammatory pathways rather than by directly modulating plasma lipids [23].

## 5. Fatty Acids (FAs)

FAs are the basic building blocks of more complex lipids. Triglyceride (TG) is the storage form of FAs and degrades via β-oxidation while releasing energy for ATP production [4]. The major categories of FAs are saturated, trans, monounsaturated and polyunsaturated FAs. FAs can be classified as saturated versus unsaturated based on the number of double bonds. There are no double bonds in SFAs which tend to be solid at room temperature, whereas unsaturated ones contain at least one (monounsaturated, MUFA), or two or more (polyunsaturated, PUFA) double bonds. FA compositions of natural foods are inherently variable; there are higher SFAs in meat and dairy products, while fruits and vegetables contain predominantly unsaturated FAs. Trans FAs are made either by the ruminal and intestinal bacterial metabolism or the hydrogenation of multiple unsaturated FAs from vegetable oils; they are hypercholesterolemic and are linked to an adverse outcome with high risk of cardiovascular diseases [24]. SFAs are considered the most harmful of all FAs, being capable to accelerate the development of atherosclerosis in the setting of insulin resistance and inflammation [14]. The brain is highly enriched in LCPUFAs-DHA (22:6n-3) and AA (20:4n-6). In the brain, PUFAs are mostly incorporated into phospholipids of neural membranes to influence membranous fluidity, signal transduction, gene transcription, and protect against neuronal apoptosis and death [25]. PUFAs act as precursors for biosynthesis of the lipid mediators which dominate the inflammatory response. N-6 FAs are precursors of eicosanoids including prostaglandins, thromboxanes, leukotrienes, lipoxins, resolvins, and eoxins. Therefore, the dietary n-3/n-6 PUFA ratio can influence the FA composition of membranous phospholipids, which are metabolized to lipid mediators which may have detrimental (pro-inflammatory effects of AA derivatives), beneficial (anti-inflammatory, neuroprotective and antioxidant effects of DHA metabolites) or neuromodulatory effects (AA-derived endocannabinoids) [26]. Alterations in the FA composition of erythrocyte also occurs in early stage of AD, prior to cognitive impairment. Compared to those with low neocortical β-amyloid load, individuals with higher β-amyloid load had elevated plasma AA and lower docosapentaenoic acid (DPA) [27].

Free fatty acids (FFAs), especially cortical elevations of FFAs, induce the assembly of amyloid and tau filaments in vitro. Although greater stimulation is usually associated with unsaturated FAs, all long-chain FAs enhanced assembly to some extent [28]. Studies testing six unsaturated FAs, including linoleic acid (LA), AA, α-linolenic acid (ALA), DHA, eicosapentaenoic acid (EPA) and oleic acid (OL), showed that all these unsaturated FAs were positively associated with neuritic plaques and NFT burden and negatively correlated with cognitive performance. In brain regions vulnerable to AD pathology—the middle frontal and inferior temporal gyri, there were decrements in LA, ALA, and AA and increases in DHA [29]. All these 6 unsaturated FAs can directly interact with Aβ_40_ and Aβ_42_ peptides and display excellent anti-aggregation properties by preventing amyloid fibril formation, especially OA and DHA [30]. However, when investigating the role of different unsaturated FAs in modulation of neuroprotective α-secretase-cleaved soluble APP (sAPPα) secretion and cell membrane fluidity, only AA, EPA and DHA with four or more double bonds are capable of increasing membranous fluidity and sAPPα secretion, whereas stearic acid (SA, 18:0), LA, ALA and OA cannot [31].

### 5.1. N-3 Fatty Acid: Docosahexaenoic Acid (DHA)

DHA is an n-3 FA and the most abundant PUFA in all brain regions [32]. DHA is derived from α-linolenic acid (ALA), forming EPA in the process. Before birth, the concentration of FAs reaches a plateau, but DHA is an exception, which continues to increase with rapid accumulation just prior to synaptogenesis [33]. Sources of DHA for humans include dietary sources such as fish, as well as DHA production in the liver [34]. The liver synthesizes 1.8 to 36 folds of DHA from ALA and EPA which are shorter chain n-3 FA precursors than the requirement of human brain [35]. The DHA level in the brain depends on the liver’s metabolic ability of diet-derived n-3 FAs in conditions of low dietary consumption of n-3 FAs such as Western diets [34].

With increasing age, there is a progressive decline of DHA level. While normal aging results in overall brain atrophy, lower DHA level is associated with increased hippocampal atrophy [36]. AD patients have decreased DHA level throughout the brain including disease resistant regions but the most prominent reduction lies in the hippocampus. According to the mini-mental state examination (MMSE), DHA content has a positive correlation with dementia [37]. Livers from AD patients also contain lower levels of DHA but higher levels of short-chain n-3 precursors including tetracosahexaenoic acid (THA), suggesting a defect in the bioconversion of THA to DHA through the peroxisomal β-oxidation, which is the last step of DHA synthesis [38]. However, some studies have shown no significant difference in DHA content between erythrocytes or the brain tissue of AD and control individuals [39,40].

In the Framingham study, subjects with the top quartile of plasma DHA level experienced a 47% lower risk of dementia [41]. In patients with mild cognitive impairment (MCI), a reduction in cognitive decline rate and better cognition were shown in the n-3 FA-treated group compared to the group treated with placebo but there was no effect in those with AD [42]. In mice, a DHA-enriched diet treatment for 8 weeks either delayed cognitive decline [43] or enhanced learning ability in mice treated longer for 7 months [44]. Multiple effects of DHA have been shown to antagonize AD pathogenesis, including increasing cerebral blood flow, decreasing Aβ deposition and tau phosphorylation by inhibiting presenilin 1 (PS1) and c-Jun N-terminal kinases (c-JNK), reducing activities of β-and γ-secretase while enhancing APP cleavage by α-secretase, increasing dendritic spine densities and restoring synaptic function in the hippocampus [45,46,47]. Since n-3 FAs are precursors for SPMs, n-3 FA supplementation in patients with MCI increased the production of SPM resolvin D1 [48] and shifted macrophages from highly proinflammatory M1 to an intermediate M1-M2 phenotype in favor of phagocytosis of Aβ [49]. While many studies support the beneficial cognitive effects of DHA, others including two double-blind randomized control trials (RCTs) reported no effect [42]. Moreover, although PUFAs are generally viewed as neuroprotective, peroxidative damage tend to affect their double bonds [50].

Among the four n-3 and n-6 FAs, DHA was oxidized to the greatest extent, followed by EPA, AA and LA. Thus, long-term treatment with DHA should be considered cautiously. Recently, it was found that deuterium-reinforced PUFAs (D-PUFAs) are more resistant to reactive oxygen species-initiated chain reaction of lipid peroxidation than normal hydrogenated PUFAs (H-PUFAs), with obviously decreased prostaglandin F2α and F2-isoprostanes in the cortex and hippocampus compared to H-PUFAs [51]. Although APP/PS1 AD mice fed D-PUFA showed lower lipid peroxidation products and Aβ_40_/Aβ_38_ production in the hippocampus compared to those fed H-PUFA, there was no change in learning and memory deficits [52].

### 5.2. N-6 Fatty Acids: Linoleic Acid (LA) and Arachidonic Acid (AA)

AA and its precursor LA are n-6 FAs. Patients with MCI and AD had elevated levels of AA but reduced levels of LA compared with healthy control and the level of LA decreased progressively from healthy control to MCI to AD patients in one study [53]. Activation of the AA cascade leads to an increase in Aβ and causes impairment in working memory induced by interleukin-1β [54]. Mice fed a diet containing 2% AA for 21 weeks had increased Aβ production and deposition [55]. AA is converted and metabolized by the enzyme 5-lipoxygenase (5-LO) to leukotrienes and by cyclooxygenase (COX) to prostaglandins and thromboxanes, all of which are associated with proinflammatory effects [56]. The 5-LO enzymatic pathway is upregulated in AD. Overexpression of 5-LO results in elevated Aβ levels whereas 5-LO inhibition results in reductions of Aβ and γ-secretase [57].

### 5.3. N-9 Fatty Acid: Oleic Acid (OA)

OA, an n-9 FA and the most abundant dietary FA, is deemed protective against AD in most studies. OA was decreased in the frontal cortex and hippocampus of AD brains [58]. The benefits of Mediterranean diet such as olive oil, which is rich in OA, are highlighted and protective against onset of AD and age-related cognitive decline [59]. OA supplementation reduced secreted Aβ and ameliorated amyloid formation in cell and animal models of AD [60]. AD patients had increased prolyl endopeptidase (PEP) activity related to their amnesia and OA demonstrated the highest PEP inhibitory activity among the unsaturated FAs [61]. Moreover, one study showed that OA leads to increased γ-secretase activity and increased PS1 and Aβ in transfected cells [62].

### 5.4. Combination of n-6 and n-9 Fatty Acids

Amtul et al. studied the effects of n-6, n-9 FAs or combination on Aβ production in vitro. LA supplementation significantly increased the Aβ_40_ levels in C-99 transfected COS-7 cells, and AA supplementation increased the levels of total Aβ including Aβ_40_ and Aβ_42_; whereas cells supplemented with OA resulted in a decrease of Aβ_40_, Aβ_42_ and total Aβ levels. Therefore, increased consumption of a n-9 FA (OA)-rich diet with reduced intake of n-6 FAs (LA and AA) may be linked to a lower risk of AD. Incubation of APP695 transfected COS-7 cells with a mixture of LA-OA-albumin or LA-OA-AA-albumin strongly enhanced the total Aβ levels by nearly two-fold in a concentration-dependent manner, revealing that lipid mixtures have additive effects on Aβ production which seem to be powerful than the effects of individual lipids. This experiment emphasizes the importance of studying lipids complex instead of single lipids regarding their effects on amyloidosis [63].

### 5.5. Dietary Fatty Acids

Of all the different species of FAs, most studies support that increased risks of AD and cognitive decline are correlated with higher saturated fatty acids (SFAs) in dietary intakes [64]. According to Chicago Health and Aging Project (CHAP), risks of AD in persons in the upper quintile of saturated-fat intake was 2.2 fold compared with those in the lowest quintile, whereas the risk was 2.5 fold for trans-fat [65]. Compared with soy oil-based diet, there were increased cerebral Aβ levels in transgenic mice fed a westernized diet containing 40% SFA for 4 months, whereas Aβ levels in persons with DHA-supplemented diets were lower than those with soy oil-based diet [66]. A MUFA-enriched diet is often known as the Mediterranean diet, which is advocated due to its association with delaying age-related cognitive decline and reducing risks of AD [64]. However, dietary intake of MUFAs was often consistent with SFA and trans FAs in dietary intakes. Studies investigating the effects of dietary lipids and AD in the real world are frequently confounded by the different compositions of FA, composing both beneficial and harmful FAs, leading to negative findings [64].

## 6. Gut Microbiota and the Gut-Brain Axis

Short-chain FAs (SCFAs) are FA with a chain length ranging from one to six carbon atoms produced mainly by colonic bacteria during the anaerobic fermentation of dietary fiber and undigested complex carbohydrates [67]. SCFAs such as acetate, butyrate, and propionate can permeate the BBB or exert their effects on the brain through the gut-brain axis [68]. Butyrate has neuroprotective effects as a histone deacetylase inhibitor through G protein-coupled receptors (GPCRs) signal pathways and anti-inflammatory signaling [69]. It is able to improve hippocampal histone acetylation and upregulate the genes expression associated with learning processes [70]. In an AD mouse model, sodium butyrate treatment facilitated learning and memory function, reduced amyloid plaque deposition and restored dendritic spine density in hippocampal neurons [70,71]. *Acetate* can affect microglia and decrease BBB permeability [72] and was found to be downregulated in AD *Drosophila* [73]

## 7. Ketogenic Diet (KD) and Ketone Bodies (KBs)

A high-fat and low-carbohydrate diet is termed KD. The metabolic pathway produces two main KBs: β-hydroxybutyrate (β-HB) and acetoacetate (AcAc). A third type of ketone, acetone, is produced by the enzymatic decarboxylation of AcAc and is mainly excreted on breath [74]. KBs can act as substrates for production of acetyl-CoA, bypassing glycolysis to enter the tricarboxylic cycle. KBs represent a normal response to hypoglycemia (e.g., fasting, higher levels of exercise, diabetes or neuropathological conditions) as an alternate fuel for the brain [75]. Following 2–3 days of KD, ketogenesis generally takes place in the mitochondrial matrix of hepatic cells. The KBs can pass the BBB via the monocarboxylic acid transporter 1 (MCT1) and then enter neurons in the brain [76]. 60–80% of dietary energy is provided by LCFAs (14–22 carbons) from classic KD. An alternative KD containing medium-chain triglyceride (MCT) was developed, with only about 45% of energy coming from medium-chain fats, thus allowing a greater carbohydrate component and better patient tolerance. The MCT-based KD is composed of about 60% octanoic acid (8-carbon FA) and 40% decanoic acid (a C10 FA) which can be metabolized to MCFAs (6–12 carbons) [77]. MCT-KD is more effective than the classic KD for two reasons: (1) direct absorption from gut into the portal vein whereas LCFAs re-esterification incorporate into chylomicrons which are absorbed via the lymphatic system and pass through the peripheral circulation before reaching the liver, and (2) no need of carnitine-dependent activation for β-oxidation as compared to LCFAs, which requires activation by carnitine to a coenzyme A before accessing the mitochondria. Octanoic and decanoic acid also have cognition-enhancing properties which are not related to KB production [78]. Reduced glucose metabolism was observed in AD patients’ brain decades before the onset of disease. In contrast, metabolism of KBs is not altered, at least in early stages of AD [79]. Therefore, KBs seem to be an alternative fuel in the brain of AD patients during hypoglycemia.

KD in AD transgenic mice resulted in reduced total Aβ levels in the brain compared with controls [80]. The addition of β-HB or Aβ or a combination to cultured rat hippocampal cells showed that β-HB reversed Aβ toxicity by doubling the number of surviving cells and increasing cell size and neurite outgrowth compared with cells exposed to Aβ only, suggesting that KBs may also act as neuronal growth factors [81]. Another probable neuroprotective mechanism of KBs is associated with their capability for upregulation of mitochondrial biogenesis [82], which improves oxidative phosphorylation and ATP generation in the brain. KBs have anti-oxidant action through increasing mitochondrial glutathione levels [83] and glutathione peroxidase activity [84]. Animals fed KD showed a reduction of free radicals by improving the efficiency of respiratory chain complex in the mitochondria [85]. Besides, KD can improve the vascular function of the brain, increase the growth of beneficial gut microbiota (*Akkermansia muciniphila* and *Lactobacillus*) and enhance metabolic profile, altogether attenuating the disease process of AD [86].

In humans, a randomized and double-blind trial administering MCT-KD to subjects with mild and moderate AD showed improved memory and cognitive function [87,88] but the beneficial effects excluded APOE ε4 carriers [87]. After using MCT-KD for 3 months followed by a 1-month washout; during the diet treatment, the mean ADAS-cognitive subscale score improved, then reverted to baseline after the washout [89]. Therefore, whether long-term KD is required for AD patients to maintain memory requires further investigations. However, KD application for the elderly arouses concerns since people with neurodegenerative diseases have risks of malnutrition and KD might lead to a reduced appetite and be accompanied by gastrointestinal side effects, exacerbating any nutritional deficits [90].

## 8. Specialized Pro-Resolving Lipid Mediators (SPMs)

As mentioned previously, aging is a process associated with inflammation. AD is a disease with exaggerated inflammation and impairment in inflammatory resolution is seen in AD patients [16]. Resolution of inflammation is regulated by a family of lipid mediators called specialized pro-resolving mediators (SPMs), which harbor anti-inflammatory properties for restoring inflammatory resolution and homeostasis. The major types of SPMs are resolvins, lipoxins, protectins and maresins. SPMs are produced by PUFAs-AA (lipoxins precursor), EPA (E-series resolvins precursor) and DHA (D-series resolvins, protectins and maresins precursors) [91]. The transition from inflammation to resolution phase is initially characterized by increases in AA-derived lipoxins and decreases in pro-inflammatory prostaglandins and leukotrienes, subsequently leading to increases in SPMs derived from n-3 FAs [92]. 

There are lower levels of SPMs in the hippocampus of AD patients. Post-mortem examination of AD patients showed reduced lipoxin A4 (LXA4) in the CSF and hippocampus compared with non-AD individuals. LXA4 and resolvin D1 (RvD1) levels in the CSF correlated with MMSE score [93]. In the entorhinal cortex, the expression of maresin 1 (MaR1), protectin D1 and resolvin D5 decreased in AD patients compared to age-matched controls. MaR1 and RvD1 down-regulated Aβ_42_-induced inflammation in human microglia and enhanced microglial phagocytosis with uptake of Aβ [48,94]. 5xFAD mice expressing APP and PS1 with multiple FAD mutations had lower SPMs in the hippocampus compared to wild types. After treatment with SPMs—resolvin E1 (RvE1) or LXA4 alone or in combination, the 5xFAD mice had a restored level of SPMs, reversed inflammatory process, and reduced neuroinflammation associated with decreased Aβ pathology [95]. Aspirin-triggered LXA4 treatment led to reduced NF-κB activation, pro-inflammatory chemokines and cytokines as well as elevated levels of anti-inflammatory IL-10 and transforming growth factor-β. Moreover, microglia were shifted to a phenotype with improved phagocytosis that promoted Aβ clearance and cognitive function [96].

## 9. Lipid Peroxidation: Isoprostanes (IsoPs), Neuroprostanes (NeuroPs)

Lipid peroxidation is the mainly manifest of oxidative stress in the CNS because of its high content of PUFAs, which are sensitive to reactive-oxygen species attack [97]. The peroxidation by-products of cerebral lipids include F_2_-isoprostanes (F_2_-IsoPs) and isofurans (IsoF), F_4_-neuroprostanes (F_4_-NeuroP) and neurofurans (NeuroF), F_2_-dihomo-isoprostanes (F_2_-dihomo-IsoP) and dihomoisofurans (dihomo-IsoF), 4-hydroxy-trans-2-nonenal (4-HNE), 4-hydroxy-2-hexenal (4-HHE), acrolein, and malondialdehyde (MDA) [98]. IsoPs or isoprostanoids are prostaglandin-like compounds, among which F_2_-IsoPs are produced by peroxidation of AA [99]. Increased F_2_-IsoPs were found in the brain and CSF of AD patients [100]. F_2_-IsoPs level in the CSF correlates with disease progression and the increase can be differentiated from normal controls with 100% accuracy [101]. Therefore, elevated F_2_-IsoPs can serve as an early biomarker of lipid peroxidation in AD patients even before Aβ depositions [102]. Lowering brain F_2_-IsoPs levels caused a significant decrease in Aβ deposition and plaque formation in the βAPP/PS1 mice [103]. Furthermore, supplementing aluminum in the mouse’s diet increased brain F_2_-IsoPs formation and led to accelerated AD phenotypes [102].

F_4_-NeuroPs are derived from oxidation of DHA; their expressions are higher in the CSF and brain of AD patients [104]. In brains of subjects with MCI and AD, the levels of F_4_-NeuroP, IsoP 8,12-iso-iPF2α-VI, HNE, MDA and acrolein were also increased [11,105]. Therefore, oxidative damage and lipid peroxidation are early events in AD [106]. Using ultra-performance liquid chromatography coupled to mass spectrometry (UPLC-MS/MS) method for the simultaneous determination of 17 lipid peroxidation biomarkers in urine samples, 17(RS)-10-epi-SC-Δ^15^-11-dihomo-IsoF, PGE_2_, NeuroP, IsoP and IsoF showed differences between patients with mild AD and control, which seems promising as potential early AD biomarkers due to their easy accessibility compared to CSF [107]. However, further investigations are required as a previous study showed that levels of F_2_-IsoPs and F_4_-NeuroPs in the plasma and urine do not correctly reflect levels in CNS of AD patients [104].

## 10. Glycerolipids

### 10.1. Glycerolipids: Triglyceride (TG)

TGs are the most predominant glycerolipids. Hyperlipidemic obese subjects treated with four to six months of lipid-lowering agent gemfibrozil had better cerebral perfusion and cognitive performance [108]. Animal studies also confirm a causal relationship between TGs and cognitive impairment, which possibly act by impaired maintenance of long-term potential through the N-methyl-d-aspartate (NMDA) component in the hippocampus, and the effects could be reversed by gemfibrozil as well [109]. However, using untargeted lipidomic analysis, Proitsi et al. found associations between low-chain and very-low-chain triglycerides (LCTGs/VLCTGs) and AD, but there were no differences in serum TG between controls and AD patients [110]. TGs were also unaltered in MCI subjects [111].

### 10.2. Glycerolipids: Monoacylglycerol (MAG) and Diacylglycerol (DAG)

MAG and DAG are elevated in the frontal cortex and plasma early in the course of AD, the MCI state [111,112]. The increase of DAG was not only observed in the cortex, but also in the white matter of MCI individuals, which could be linked to phospholipase degradation of phosphatidylethanolamines (PE) [113]. Aβ_1–42_ peptide could enhance production of DAG via phospholipase D (PLD) in SH-SY5Y neuroblastoma cells. The PLD-produced DAG participates in a decrease of soluble amyloid precursor protein α (sAPPα) secretion mediated by Aβ [114].

Monoacylglycerol lipase (known as MAGL) is the primary enzyme that catalyses the hydrolysis of MAG to FFA and glycerol and metabolizes the endocannabinoid 2-arachidonoylglycerol (2-AG) in the brain [115]. Inactivation of MAGL suppressed β–secretase 1 (BACE1) expression and reduced Aβ production and accumulation in a mouse model of AD. MAGL inhibition also exerted anti-inflammatory effect and neuroprotective response, thence improved synaptic functions and cognitive skills in AD animal models [115]. MAGL inhibitor JZL184 decreased the pro-inflammatory response of microglia and reduced the total Aβ load in the APdE9 transgenic mouse model [116]. JZL184 treatment for Ts65Dn mice, a Down syndrome mouse model, also showed an increase in hippocampal long-term potentiation and decreased levels of Aβ_40_ and Aβ_42_. URB602, a selective MAGL inhibitor, exhibited an effect of neuroprotection on homocysteine-mediated impairment by reducing cyclooxygenase-2 (COX-2) elevation and ERK1/2 and NF-κB phosphorylation as well as decreasing IκB-α degradation [117].

## 11. Glycerophospholipids

### 11.1. Glycerophospholipids (Phosphoglycerides)

Glycerophospholipid is the major type of lipid defining the cell membranes. The major phospholipid in human brain is ethanolamine phosphoglyceride (35.6%) [118,119] and its predominant form is the ethanolamine plasmalogen (PlsEtns), whereas phosphatidylethanolamine (PE) makes up the remaining amount. Phosphatidylcholine (PC) is the principal form of choline phosphoglycerides in the human brain, accounting for 32.8% [118]. Except in muscles, ethanolamine plasmalogens are 10-fold greater than choline plasmalogens.

In the brain of AD patients, PC and PE were significant decreased and phospholipid deacylation products glycerophosphocholine were increased in the frontal, primary auditory and parietal cortices [120]. The reduction in PE (P-16:0/20:40) and PE (P-16:0/22:6) correlated with AD severity. Plasmalogens can reduce γ-secretase activity and their depletion results in incrased Aβ [121]. Depletions of PI (16:0/20:4), PI (16:0/22:6) and PI (18:0/22:6) were involved in facilitating Aβ_42_ biogenesis. In AD, an enhancement in the ethanolamine-containing plasmalogens and platelet activating factor (PAF) precursors hydrolysis results in accumulation of PlsEtns and *PAF* metabolism products [i.e., PC(O-16:0/2:0) and PE(P-16:0/0:0)], which accelerates tau pathology, enhances vesicular release, and signals neuronal loss [122]. PAF is an ether-glycerosphospholipid important for immune cell activation [123]. 

### 11.2. Glycerophospholipids: Ethanolamine Plasmalogen (PlsEtn) Deficiency and Peroxisomal Dysfunction

PlsEtn represents over 50% of the total ethanolamine phosphoglycerides in neurons and over 85% of the content in myelin [124]. Relative to PE, a selective defect of PlsEtn was determined in AD patients’ brain and the lipid deficiency displayed anatomic specificity, especially in the temporal cortex [125]. In human brains, there was an up to 40% reduction of plasmalogen in white matter of frontal, parietal, and temporal regions at a very early stage of AD, while a 30% reduction in grey matter occurred at an advanced stage and this reduction correlated with disease severity [126]. The deficiency of PlsEtn precedes the clinical manifestation of dementia by many years and the degree of reduction correlated with disease severity [127]. However, another study demonstrated that PlsEtn and PE were decreased in the grey matter of young and old dementia patients but not altered in the MCI group compared to cognitively intact subjects [113], implying that alterations in plasmalogen are unlikely to represent an initiating event in the transition from MCI to dementia. Plasmalogens need intact peroxisomes for their biosynthesis, as such, their reduction in tissues are in agreement with peroxisomal dysfunction [4]. The reduction of PlsEtn and PC as the consequences of peroxisomal dysfunction in AD patients were accompanied by the accumulation of C22:0 and VLCFAs (C24:0 and C26:0), substrates for peroxisomal β-oxidation in the cortical regions with stages V-VI pathology compared with those at stages I-II [128]. A loss of peroxisomes has been demonstrated in neuronal processes with abnormal tau phosphorylation by confocal laser microscopy [128]. Fourier transform infrared microscopy characterized the senile plaques and their immediate surroundings as the presence of oxidized lipids [129]. Therefore, oxidative stress may be causal for the pathogenesis and progression of AD and that PlsEtn deficiency supports the oxidative stress hypothesis in AD. The anti-apoptotic action of PlsEtns in the brain was indicated by the inhibition of hippocampal neuronal cell death, which was associated with suppression of caspase-3 and caspase-9 cleavages and enhanced phosphorylation of AKT and ERK signaling [130]. Intraperitoneal injection of plasmalogen and lipopolysaccharide (LPS) for 7 days to C57/6 J mice attenuated neuroinflammation and abolished Aβ accumulation in the hippocampus [131]. A multicenter, randomized, double-blind and placebo-controlled trial in MCI and mild AD patients aged 60 to 85 years received 1mg/day of PlsEtn purified from scallops for 24 weeks. In the treatment group, mild AD patients revealed a smaller reduction of plasma PlsEtn and improved cognitive functions compared to the placebo group [132].

### 11.3. Glycerophospholipids: Phosphatidylcholine (PC)

Three PCs were found significantly diminished in AD: PC (16:0/20:5), PC (16:0/22:6) and PC (18:0/22:6) [133]. Mapstone et al. conducted a 5-year observational study in healthy elderly patients and identified ten metabolites, comprising seven PCs, one lysophophatidylcholine and two acylcarnitines that were depleted in the plasma of the MCI or AD and the depletion could identify (with accuracy above 90%) cognitively normal individuals who, on average, will convert to MCI or AD within 2–3 years [134]. A 73% decrease of choline plasmalogen was found in the postmortem prefrontal cortex (Brodmann area 9) of AD patients compared to the non-AD control [135]. Cytidine-5-diphosphate-choline (CDP-choline) participates in the phospholipid metabolism pathway incorporating free choline into phosphatidyl-choline and choline plasmalogens. CDP-choline treatment for one month to early-onset AD patients resulted in significant improvement in mental performance [136]. Although most studies reported a reduction of PC levels in AD, contradictory findings have also been reported. Proitsi et al. found that the lipids most strongly associated with AD are PC 40:4 and PC 36:3, both of which were increased in AD [110]. Kennedy et al. combined gene expression profiling with a genome-wide screen and found that PC(O-16:0/2:0) was elevated in AD [137]. An increase in CSF PC was observed in AD compared to control brains. During normal aging, the plasma levels of lysophosphatidylcholine, choline plasmalogen, and lyso-PAF increase significantly; similar but more pronounced changes in these choline-containing phospholipids were observed in AD patients [138].

### 11.4. Phospholipase

Phospholipases catalyze the hydrolysis of phospholipids to liberate PUFA from the cell membrane, which can be divided into four different classes based on the cleavage site. Phospholipase A2 (PLA2), which releases AA, is implicated in AD [139]. The proinflammatory enzyme PLA2 circulates in plasma with its active form as a complex along with low-density lipoprotein (LDL) and high-density lipoprotein (HDL). The level of plasma lipoprotein-associated PLA2 was higher in AD patients [140]. Secretory PLA2 activity in the CSF was also significantly higher in AD [141].

Phosphoinositide (PI) signal transduction pathway is involved in regulation of various cell functions, such as cell growth, cell cycle control, apoptosis, membrane trafficking, cytoskeleton regulation, hormone secretion, neurotransmitter signal transduction, ion channel activity, cell and tissue polarity, and Ca^2+^ regulation in the nervous tissue. PI-specific phospholipase C (PLC) is one of the key enzymes involved in neurotransmission [142] and is linked to several brain disorders including AD [143]. PLCs have 6 isoenzymes (β, γ, δ, ε, ζ, η) [144]. An earlier study showed that PI-specific PLC activity was not altered in AD [145] but immunostaining against one of the PLC isozymes, PLC_δ_ demonstrated that this enzyme accumulated in NFTs. Chromatofocusing profiling showed a significant reduction of PLC _γ1_ and a concomitant increase of phospholipase C_δ1_ activity in AD brains compared with controls, suggesting that the involvement of PLC isozymes in AD is different [146]. PLC also plays a crucial role in regulating intracellular Ca^2+^. PLC_η_ is highly abundant in brain regions associated with cognition and memory and involves in the modulation and amplification of Ca^2+^ signals [147].

Phospholipase D (PLD) is the enzyme catalyzing the hydrolysis of PC to phosphatidic acid (PA) and choline; the latter as an acetylcholine synthesis precursor. Early pathological changes of AD are characterized by cholinergic dysfunction with neuronal loss, starting from cholinergic neurons. The PLD pathway has been demonstrated for an important role in amyloidogenesis [148]. Overexpression of APP in mouse P19 embryonic carcinoma cells increased PLD activity [149]. Aβ stimulated PLD activity in cultured neuronal cells, suggesting that PLD activation participated in Aβ-induced neurotoxicity and AD [150]. PLDs have three isoforms - PLD1, PLD2 and PLD3. PLD1 modulated the down-regulation of APP processing, likely by mediating PS1 activity [148]. The synaptosomes from AD patients’ temporal cortex and hippocampus showed an increase of PLD1 expression. The inhibition of PLD1 blocks the synaptic dysfunction in the hippocampus of 3xTg-AD mice in early-stage (Aβ driven) and late-stage (Aβ and tau driven) [151]. Expression of PLD3 is elevated in brain regions which are vulnerable to AD, including the hippocampus and cortex [152]. Carriers of PLD3 coding variants have a 2-fold increase in late-onset AD risk [152,153]. PLD3 overexpression significantly down-regulates intracellular APP, extracellular Aβ_40_ and Aβ_42_, whereas the knockdown of PLD3 increases extracellular Aβ_40_ and Aβ_42_ [152]. However, several follow-up genomic studies failed to replicate the impact of PLD3 risk variants in AD [154,155]. More validation studies are needed to clarify the relevance of PLD3 in AD pathogenesis.

### 11.5. Glycerophospholipids: Cardiolipin

Cardiolipin is a phospholipid mainly found in the inner mitochondrial membrane responsible for the maintenance of fluidity and activity of mitochondrial electron transport chain enzymes [156]. Normal aging brains exhibit decreased cardiolipin and increased cardiolipin peroxidation, which results in reduced complex I activity of the mitochondrial electron transport chain [157]. Reduction of cardiolipin in synaptic mitochondrial membranes was reported in the brains of AD patients [158]. Liposomes-containing cardiolipin promotes ability of nerve growth factor to cross the BBB and increases the neuronal survival by reducing Aβ_1–42_ neurotoxicity. However, subsequent studies are required to confirm their clinical application [159].

## 12. Sphingolipids

There are two major classes of sphingolipids according to the polar head group: phosphosphingolipids and glycosphingolipid. The former includes sphingomyelin (SM) and the latter includes cerebrosides and gangliosides [160]. Sphingolipids promote the metabolism of APP via apoptosis, calcium homeostasis, tau phosphorylation, acetylcholine biosynthesis and amyloidogenic pathway [161].

### 12.1. Sphingolipids: Sphingomyelins (SMs) and Sphingomyelinase (SMase)

SM is the most abundant sphingolipid in the brain and is found abundantly in myelin sheaths [162]. Increased SM was found in the brain tissue of AD subjects using 31P nuclear magnetic resonance study. Metabolomic assays in AD brains found that higher concentration of SMs were associated with severity of AD pathology and increased risk of abnormal cognition, including 3 SM with acyl residue (SM C16:0, SM C16:1, SM C18:1) and 1 hydroxysphingomyelin with acyl residue (SM (OH) C14:1) [161]. However, the changes of SMs in AD were inconsistent among studies. SMs are important components of lipid rafts where they act as γ-secretase inhibitors and decrease the synthesis of Aβ_40_ and Aβ_42_ peptides [163].

Sphingomyelinase (SMase) is an enzyme catalyzing sphingomyelin to ceramide by hydrolysis. Based on the pH, SMase is classified into three forms: acid sphingomyelinase (ASM), neutral sphingomyelinase (NSM), and alkaline sphingomyelinase (alkSM) [164]. According to the studies on mouse model, intracerebral injection of Aβ promoted SMase and ceramide levels [165]. Aβ activated NSM but not ASM; NSM inhibition attenuated Aβ-induced oligodendrocytes death by 3-O-methyl-sphingomyelin or by genetic knockdown using antisense oligonucleotides [166]. Upon the genetic expression levels, ASM and NSM2 were upregulated in AD [167]. ASM is a lysosomal glycoprotein. According to AD patients and mice data, enhanced activity of ASM was noticed in plasma, fibroblasts, and brain contributing to defective autophagic degradation due to lysosomal depletion. In a mouse model of familial AD (APP/PS1), partial genetic inhibition of ASM (ASM (+/-)) improved the autophagocytic defect by restoring lysosomal biogenesis, resulting in a reduction of Aβ deposition and improvement of memory impairment. Similar effects were noted after pharmacologic restoration of ASM to the normal range in APP/PS1 mice [168].

### 12.2. Sphingolipids: Ceramides and Exosomes

Ceramides play an important role in sphingolipid metabolism and as second messengers of lipids. Ceramides can be generated from SM hydrolysis or synthesized *de novo* in the endoplasmic reticulum. They are related with inflammation and neuronal apoptosis, particularly the MAP kinase (MAPK) and AKT pathways [169].

Lipidomic studies found increased ceramide levels in AD brains [167,169,170], particularly ceramides Cer16, Cer18, Cer20, and Cer24 [167]. There were lots of saturated ceramides Cer(d18:1/18:0) and Cer(d18:1/20:0) in senile plaques [171]. Increased levels of ceramides were also found in the CSF [172] and serum of AD patients. High baseline plasma levels of Cer 16:0 and Cer 24:0 are consistent with increased risk of AD in older women [173], and higher Cer 22:0 and Cer 24:0 levels suggest the loss of hippocampal volume and cognitive decline. Han et al. observed an enhancement of ceramide in the early AD stages, while its concentration reduced with disease severity [174]. Ceramides enhance Aβ formation by stabilization of β-secretase enzyme BACE1 which modulates APP processing. The Aβ formation induces increased levels of ceramide through catalyzing the breakdown of SM to ceramide by SMase as a positive feedback loop [169]. 

Exosomes are nanoparticles with a diameter in the range of 40–150 nm that are generated by inward budding of multivesicular bodies (MVBs) and secreted from cells when MVBs fuse with the plasma membrane. Extracellular vesicles (EVs) are exosomes that are enriched with ceramides as well as other gangliosides [175]. Recent research found that increased exosome secretion in the brain is able to play an active role in the progression of AD [176] because exosomes can accelerate Aβ aggregation [177]. Ceramide triggers budding of exosomes into multivesicular exosomes while exosome secretion in neural cells can be significantly reduced by inhibiting NSM-2 [177,178]. Reducing exosome secretion in NSM2-deficient 5XFAD mice ameliorated AD pathogenesis with reduced glial cell activation, tau phosphorylation and total Aβ plaque deposition and improved cognitive function compared to 5XFAD AD model mice [179].

### 12.3. Sphingolipids: Sphingosine 1-Phosphate (S1P)

S1P is produced from the hydrolysis of ceramide and sphingosine by sphingosine kinases (SphKs), which have two isoenzymes including sphingosine kinase-1 (SphK1) and sphingosine kinase-2 (SphK2). Sphingosine 1-phosphate lyase (SPL) can degrade the S1P. Level of S1P was decreased in the AD brain [180,181]. Deficiency of SPL with accumulation of S1P impairs the degradation of APP and amyloidogenic C-terminal fragments in lysosomes as well as decreases the activity of γ-secretase, thereby reducing amyloidogenesis [182]. Sphk1 is a pro-survival signaling mediator. Glucose reload stress/glucose deprivation upregulated the expression and activity of Sphk1 in hippocampal neurons [183]. A decreased expression of SphK1 and an increased expression of SPL, with a loss of pro-survival S1P, were related to Aβ accumulation in the entorhinal cortex of AD brains [184]. Conversely, Sphk2 was upregulated in the brains of AD. A S1P transporter Spinster homolog 2 (Spns2) enhanced pro-inflammatory response of activated microglia in vivo and in vitro. Spns2 knock-out in mice ameliorated Aβ_42_-induced impairment of working memory [185]. Stimulation of S1P receptors seem promising in a few in vitro and in vivo AD models [186]. These studies suggested that sphingolipid metabolism plays a central role in AD pathology.

### 12.4. Sphingolipids: Sulfatides

Sulfatides are essential component of myelin synthesized almost entirely in oligodendrocytes and participate in the stabilization of oligodendrocyte membranes. An abrupt decrease of sulfatide was observed in the early stages of AD, whereas a very little change in its concentration was observed until the advanced stages [187]. While PI remains constant, sulfatides in the CSF also decrease in the early stages of AD. It was proposed that the sulfatide: PI ratio may be the marker for early-stage diagnosis of AD [188]. ApoE mediates metabolism/trafficking/homeostasis of sulfatides in APP transgenic mice and modulates sulfatide levels [189]. ApoE4 overexpression caused a 60% decrease in sulfatide concentration in the brain of transgenic mice. Sulfatides enhance Aβ binding to ApoE-associated particles and enhance Aβ uptake, which leads to Aβ accumulation in lysosomes [189].

### 12.5. Sphingolipids: Gangliosides

Acidic glycosphingolipids which are referred to as gangliosides contain one or more sialic acid(s) connected to the inner galactosyl residue in their carbohydrate moiety. Gangliosides are most abundant in the CNS, and they preferentially cluster in lipid rafts and outer plasma membranes. Gangliosides can be classified into series of the 0 (or asialo), a, b and c, according to the number of sialic acid residues.

There was a significant reduction of ganglio-series gangliosides (GM1, GD1a, GD1b and GT1b) in the basal telencephalon and frontal and temporal cortex of AD brains, which probably correlates with degeneration of cortical neurons [190]. Analysis of ganglioside subtypes “a”-ganglioside (GM1 and GD1a) and “b”-ganglioside (GD1b and GT1b) indicated that “b”-gangliosides are preferentially influenced in AD individuals and consistently showed reduction among different studies [190]. Compared to the major gangliosides, simple gangliosides like GD3, GM2, GM3 and GM4 were increased in the frontal and parietal cortex of AD brains, which might correlate with an accelerated astrogliosis and/or lysosomal degradation of gangliosides during neuronal death.

Soluble Aβ expresses a high affinity in order to bind to gangliosides-containing lipid rafts under physiological conditions [191]. A distinctive ganglioside-bound form of Aβ (GAβ) was found in the AD brains due to ganglioside-mediated conformational Aβ alteration [192,193]. Gangliosides can modulate Aβ aggregation and cytotoxicity, however, Aβ bound to GM1 ganglioside exhibited the strongest Aβ seeding potential [192,194]. Depletion of glycosphingolipids using D- and L-Threo-1-phenyl-2-decanoylamino-3-morpholino-1-propanol remarkably inhibited the secretion of endogenous APP and Aβ. Conversely, the addition of exogenous brain gangliosides reversed these effects [195].

The entorhinal cortex of AD subjects showed an abundance of SM, the ganglioside GM3, lysobisphosphatidic acid and cholesterol esters, suggesting that the pathophysiology is associated with disorders of endolysosomal storage. An enhancement of GM3 and cholesterol esters was recapitulated in familial AD transgenic mouse models. The genetic ablation of phospholipase D2 fully normalizes GM3 levels and recued the synaptic and behavioral deficits. This study suggests a crosstalk between the metabolism of gangliosides, phosphatidic acid and the product of phospholipase D2, indicating an important role of the ganglioside anomaly in AD pathogenesis [112].

GM1 can protect hippocampal neurogenesis from D-galactose injury in the aging mice [196]. B-series gangliosides, especially GD3, regulate the maintenance of the neural stem cells self-renewal capacity in vitro [197]. Semisynthetic GM1 such as LIGA20, LIGA4 and PKS3 are more potent than the parent natural compounds, with faster and longer actions and are potent and efficacious antagonists of glutamate-induced neuronal death. Ala et al. used intramuscular injections of GM1 for 12 weeks whereas Flicker et al. used the same regimen for 6 weeks to treat AD patients. Although safe, the treatment offered no cognitive profit to mild-to-moderate AD patients [198].

Svennerholm et al. reported that intraventricular administration of GM1 for 1 year in early-onset AD cases halted AD progression and improved motor performance and cognitive function such as reading and feeling for language [199], implying that long-term administration of GM1 may be useful for AD patients. Matsuoka et al. reported that peripheral administration of GM1 every 2 days for 2 weeks to PS/APP mice reduced Aβ in the brain of young mice but not 6–7-month-old mice with severe Aβ burden, which suggested that early GM1 administration may reduce or prevent brain amyloidosis [200]. Yang et al. demonstrated that microinjection of GM1 into the hippocampal dentate gyrus in rat model of AD induced by Aβ1–40 injection improved learning and memory disorders via reduction of lipid peroxidation and oxidative stress due to concurrent reduction of malondialdehyde (MDA) and HNE levels in the hippocampus [201].

## 13. Cholesterol

The major sterol lipid in humans and animals is cholesterol. The main source of brain cholesterol is originated from de novo biosynthesis, since the BBB prevents any plasma lipoproteins from efficiently entering the brain [202]. Lack of cholesterol provision to neurons impairs synaptic plasticity and neurotransmission, as well as inducing tau pathology and neurodegeneration [203].

Cholesterol and cholesterol esters play important roles in amyloidogenesis [202,204]. Low expressions of cholesteryl esters and free cholesterol correlated with an increase of Aβ production and loss of neuronal membrane cholesterol caused amyloidogenesis [205]. During aging, cholesterol depletion from neurons is also associated with impaired neurotransmission, synaptic loss, enhanced tau pathology and neuronal death [206].

Conversely, cholesterol induced or exacerbated cerebral amyloidosis in animal models [66,207]. Elevated cholesterol is responsible for Aβ formation and was observed in early stages of AD patients [208]. Aβ production is mainly determined by β-secretase 1 (BACE1) levels in lipid rafts, the enzyme that cleaves βAPP to generate Aβ [204]. High cholesterol influences APP processing in various pathways including regulating all types of APP proteolytic secretases, α-, β-, and γ-secretase. Cholesterol also mediates Aβ metabolism in many aspects, including its fibrillation, transportation, degradation, and clearance processes [209]. Mouse models of familial hypercholesterolemia have increased BBB permeability and oxidative stress, so as to enhance sensitivity to Aβ-induced neurotoxicity [210]. Hypercholesterolemic diet accelerated AD pathology in animal models and caused significantly increased Aβ load [207]. Rabbits fed a 1% cholesterol for 7 months had increased cholesterol in the neurons, which was accompanied by increased BACE1 levels and accumulation of Aβ_42_ and phosphorylated tau in the hippocampus [211]. Statin, the cholesterol lowering drugs, reduced the accumulation of Aβ in the brain [212]. Another study demonstrated that cholesterol retention did not directly affect activity of BACE1, but induced βAPP clustering and rearrangement in BACE1-presenting lipid rafts and rapidly internalized into endosomes where βAPP cleavage occurred, leading to enhanced Aβ production [213].

MCI and dementia-free participants had a lower first-visit total cholesterol compared to participants with dementia [214]. Familial hypercholesterolemia patients showed an elevated incidence of MCI, which finally progressed to AD in the majority of cases [215]. Postmortem brain samples from patients with AD showed significantly lower level of HDL and higher level of LDL cholesterol [216]. A population-based study found that cholesterol in combination with hypertension in midlife elevated the risk of AD in later life significantly [217]. In contrast to most studies, in the Framingham Heart Study, hypercholesterolemia was associated with better cognitive function. Using LC/MS on plasma samples of AD, MCI and control individuals, no association between cholesterol and AD was found [218,219], and there was no difference in the frequency of AD patients and controls who were prescribed statins [110].

Different from cholesterol, oxidized cholesterol metabolites which are known as oxysterols, like 24S-hydroxycholesterol (24S-OHC) and 27-hydroxycholesterol (27-OHC), are capable of passing through the BBB and are increasingly recognized as having pivotal roles in AD [219]. Brain cholesterol homeostasis is determined by biosynthesis of cholesterol and diffusion of oxysterols between blood and brain [220]. When the level of cholesterol exceeds the physiological basis, it is converted to 24S-OHC and actively eliminated from neuronal cells due to its neurotoxicity [221]. In early AD, higher 24S-OHC levels are found in the plasma [222]. However, the level of 24S-OHC in the serum was decreased in the case of chronic and advanced stages of AD, corresponding with clinical observations showing that the effect of serum total cholesterol on the risk of dementia occurs in midlife but not in late-life. While there is a decrease of 24S−OH in late stages of AD, other oxysterols such as 27−OHC and 25−hydroxycholesterol are significantly increased [223]. High levels of 27−OHC are found in the brains and CSF of early-onset AD as well as in sporadic AD [224]. 27−OHC treatment in vitro increased tau phosphorylation and Aβ production [225,226] and caused dendritic spine loss in vitro and in vivo through the retinoid X receptor gamma (RxR_γ_) [227], although the action of 27−OHC on synaptic function and plasticity is still unknown. The *CYP27A1* gene knockout in mice could deplete 27−OHC and ameliorate memory impairments induced by a high cholesterol diet, which indicates that 27−OHC is the main contributor to the memory disorder caused by dietary cholesterol [228]. These conflicting results between cholesterol level and amyloidogenesis or AD risk suggest that brain cholesterol homeostasis is tightly regulated and both low or high levels may lead to AD.

## 14. Apolipoprotein E (ApoE)

ApoE is the main component of lipoproteins, which mediates the transport of cholesterols and phospholipids in the brain [209]. E2, E3 and E4 are the important isoforms of ApoE, each encoded by different alleles (ε 2, 3, and 4). ApoE3 is the major isoform (77–78%) in human, while ApoE4 and ApoE2 account for 14–15%, and 7–8%, respectively [229]. ApoE4 binds to very low-density lipoproteins (VLDLs) which are large and TG-rich, while ApoE2 and ApoE3 preferentially bind to small, phospholipid-rich high-density lipoproteins (HDLs). ApoE2 decreases levels of total cholesterol whereas ApoE4 enhances them.

*APOE* ε4 on chromosome 19 has been known as the most prevalent genetic risk factor of AD [209], which contributes to approximately 50% of sporadic AD [230]. ApoE2 reduces risk of AD, whereas the ApoE4 allele confers an increased risk and decreased age of onset in AD. One ε4 allele assigns a 3-fold increase in risk of AD whereas two alleles impart a 12-fold increase [231]. Individuals homozygous for the *APOE* ε4 allele have elevated cholesterol levels in the plasma and increased 24S-OHC levels in the CSF [232]. High plasma and brain AA/DHA ratios in the phospholipids were observed in familial AD mouse model expressing *APOE* ε4-carriers compared to those expressing APOE isoforms [233].

Besides, ApoE4 carriers are remarkably affected by n-3 FAs deficiency in diet. When receiving a n-3 FA-deficient diet, mice carrying ApoE4 had a greater reduction of n-3 FA levels in tissues and organs than other *APOE* mice receiving the same diet. Supplementing DHA as soon as possible could inhibit the disease progression and reverse the neurological and behavioral deficits in ApoE4 mice [234].

ApoE mediates Aβ internalization by binding on the LDL receptor-related protein to affect Aβ clearance and promote Aβ aggregation [235]. When lacking ApoE, the amount of Aβ in lipid rafts was reduced, and Aβ fibrils failed to form [236]. ApoE affects senile plaque load in an isoform- and dose-dependent fashion (ApoE4 > ApoE3 > ApoE2) [237,238]. ApoE4 promotes Aβ aggregation and deposition and impairs Aβ clearance in the brain [238,239,240].

ApoE is also a critical determinant of brain phospholipid homeostasis and the ApoE4 isoform is less effective in this process. Zhu et al. found that postmortem human brain tissues of ApoE4 carriers had a lower level of phosphoinositol biphosphate (PIP2) compared with those of ApoE3 counterparts at early stages of AD, and similar results were also found in primary neurons expressing ApoE4 alleles and in the brains of ApoE4 knock-in mice. In ApoE4 carriers, genetic knockdown of PIP_2_-degrading enzyme, the phosphoinositol phosphatase synaptojanin 1 (Synj1), restored PIP_2_ homeostasis in the brain and rescued cognitive deficits [241]. Changes in PIP_2_ secondary to increased expression of Synj1 has been mentioned in a previous study [242].

## 15. Statins

Statins inhibit 3-hydroxy-3-methyl-glutaryl-CoA (HMG-CoA) reductase, which is the regulator of cholesterol biosynthesis rate-limiting step. From a national health insurance dataset, progression of AD was statistically decreased in AD patients with early use of statin than those without [243]. Satins reduced the risk of AD to 67–73% [244]. Statins treatment to ApoE4-carrying AD patients showed less cognitive impairment over the 10-year follow-up course compared with those without treatment [245]. The Canadian Study of Health and Aging showed a positive effect of statins on decreasing incidence of dementia in persons under 80 years but not in those over 80 years [246]. It seemed to coincide with previous studies showing that reduction of cholesterol in late-life has no impact on risk of dementia. Among the studies showing beneficial effects of statins, the reduction in AD risk varies across different statins, sex, and race/ethnicity. Simvastatin and Atorvastatin seemed to have more consistent effects among different races and genders compared to pravastatin and rosuvastatin [247]. Two RCTs—the Heart Protection study (HPS) [248] and the PROSPER study [249], strongly refuted the concept that lowering cholesterol by statin prevents dementia. Two RCTs (The LEADe study using atorvastatin for 72 weeks followed by 8-week withdrawal [250] and another study using simvastatin for 24 months [251]) in mild to moderate AD patients did not show benefits on disease progression.

Statins suppress tau phosphorylation [252], decrease BACE1 and APP production [253], and can interact directly with Aβ to attenuate amyloidosis [254]. Simvastatin and atorvastatin increased the extracellular Aβ degradation of Neprilysin (NEP) on astrocytes by inducing ERK-mediated pathways [255].

The effects of statins on amyloidosis may involve pathways independent of cholesterol biosynthesis. The HMG-CoA reductase pathway, also known as the mevalonate pathway, synthesizes isoprenoids, geranylgeranyl pyrophosphate (GGPP), and isoprenoid intermediates - farnesyl pyrophosphate (FPP) in addition to cholesterol [256]. High cortical levels of FPP and GGPP significantly correlate with tau phosphorylation, NFT density and early-onset AD. hFPPS and hGGPPS mRNA expression in the cortex positively correlate with levels of HMG-CoA reductase in AD individuals but not with levels of tissues cholesterol [257]. In an AD mouse model, atorvastatin exerts anti-inflammatory effects by reducing FPP [258]. Simvastatin also ameliorated neuroinflammatory response by decreasing NF-κB, rescued oxidative damage and attenuated hippocampal cell apoptosis [253,259].

## 16. ATP-Binding Cassette (ABC) Transporters

ABC transporters are located in the plasma membrane as well as the membrane of intracellular organelles which mediate the active transport of a variety of molecules to maintain cellular homeostasis [260]. ABC transporters are key regulators of lipid homeostasis by mediating the export of cholesterol and phospholipids in the brain.

Among the ABC subfamily-A (ABCA), there are six transporters (ABCA1, ABCA2, ABCA3, ABCA5, ABCA7 and ABCA8) expression in the brain [261]. Currently, four members of the ABCA family have been reported to be associated with AD (ABCA1, 2, 5, and 7). ABCA1 modulates Aβ formation through the mediation of ApoE. ABCA2 promotes β- or γ-secretase cleavage of APP. ABCA5 inhibits the Aβ secretion and ABCA7 mediates the uptake and clearance of Aβ [7].

ABCA1 is a cholesterol transporter. ABCA1 and lipoprotein binding are important for lipid efflux. ABCA1-deficient mice had a decreased amount of cholesterol in the CSF [262]. ABCA1 loss-of-function mutation is strongly related with a higher AD risk [263]. Low expression of ABCA1 results in impaired clearance of Aβ [262,264], whereas its high expression inhibits the deposition of amyloid in the murine AD model [265]. ABCA1 deficiency increased Aβ aggregation in ApoE4 mice with an APP/PS1 transgenic background, but not in ApoE3 mice, and this result showed that the effect of ABCA1 on Aβ clearance depends on ApoE isoforms [266].

ABCA2 regulates lipid metabolism via the low-density lipoprotein receptor [267]. Microarray gene expression datasets from prefrontal cortical tissue and blood showed that overexpression of ABCA2 is seen in AD compared with controls. *ABCA2* mRNA expression and methylation are associated with risk of AD [268]. ABCA2 colocalized with Aβ [269]. Overexpression of ABCA2 in vitro in human embryonic kidney cells and N2a neuroblastoma cells was associated with increased expression of the *APP* gene through increased transcription and promotes cleavage of APP by BACE1 [269]. Knockdown of *ABCA2* altered γ-secretase processing of APP and reduced Aβ production in vitro and in vivo [270].

ABCA5 acts on Aβ production rather than its clearance. ABCA5 reduces Aβ_40_ and Aβ_42_ formation without altering mRNA and protein levels of APP, indicating that the decrease in the Aβ levels is due to modulation of APP processing [271].

GWAS has found *ABCA7* as a genetic risk factor for late-onset AD [272]. ABCA7 exports choline phospholipids and lysoPC is one of its major lipid substrates [273]. ABCA7 expression is elevated in human phagocytes, including macrophages and microglia [261] and mediates the clearance of apoptotic cells via ERK signaling [274]. Therefore, ABCA7 deletion reduced the phagocytic clearance of Aβ [275]. ABCA7 overexpression diminished Aβ deposition and improved cognitive behavior in AD mice. Meanwhile, overexpression of ABCA7 relieved the Aβ neurotoxicity by reducing endoplasmic reticulum stress and promoting cell viability [276]. Although ABCA7 seems protective against AD, expression of ABCA7 is increased in AD individuals on the contrary. It was proposed that the increase of ABCA7 observed in AD reflects an inadequate compensatory change [277]. The levels of ABCB1 are decreased [278] and its activity at the BBB is significantly compromised in AD patients [279]. ABCB1 actively mediates Aβ transport across the apical membrane of brain capillary endothelial cells by directly interacting with Aβ_40_ and Aβ_42_ [280].

ABCG2 interacts directly with Aβ [281] and promotes efflux of Aβ_40_ and Aβ_42_ across the BBB, thereby decreasing amyloid deposition in the brain. ABCG2 prevents reactive oxygen species (ROS) generation and activation of the ROS- responsive NF-κB pathway, resulting in a reduced expression of inflammatory genes. ABCG2 overexpression reduces Aβ production, which might be associated with the inhibition of a positive modulatory effect of ROS on the activity of AβPP processing enzymes [282]. Contrary, mRNA and protein expression of ABCG2 were found to be strongly upregulated in the AD brains [281]. The ABCG2 upregulation in AD may be a compensatory mechanism during oxidative stress in order to inhibit the NF-κB signaling pathway and associated pro-inflammatory responses [282].

## 17. Lipid A

Monophosphoryl lipid A (MPL) is a lipopolysaccharide (LPS)-derived Toll-like receptor 4 (TLR4) agonist that is capable of facilitating an immune response similar to LPS but much less potent [283]. In transgenic mice overexpressing APP, immunization with Aβ_42_ adjuvanted with MPL decreased the accumulation of cerebral Aβ by 60% [284]. Immunization of non-human primates with Aβ_42_ admixed with MPL resulted in a shift in the size of Aβ toward smaller species, which might facilitate removal of toxic Aβ from the brain [285].

Both in vitro and in vivo studies have shown that MPL stimulated uptake of Aβ by microglia. APP/PS1 mice with repeated intraperitoneal MPL injections had reduced Aβ production in the brain and recovery of cognitive impairment [283]. The neuroprotective nature of MPL is likely due to its ability to stimulate microglial phagocytosis of Aβ without eliciting a strong proinflammatory response [286]. Oral administration of LPS to mice could activate peritoneal macrophages [287] and enhance the phagocytic activity of Aβ_1–42_ by primary microglia via the TLR4 pathway [288]. Studies using low doses of either MPL or TLR2 agonist-Pam3Cys administered intracerebroventricularly to rats treated with Aβ_1–42_ improved their memory function; restored the impaired long-term potentiation induced by Aβ; decreased TNF-α and Aβ deposits, enhanced expression of microglial marker, arginase 1, and increased polarization of hippocampal microglia to an anti-inflammatory phenotype [289]. 

## 18. Fat-Soluble Vitamins: Vitamin A, D, and E

### 18.1. Vitamin A

Vitamin A, provitamin A carotenoid, and vitamin A derivative retinoids are considered antioxidant compounds. In AD patients, the levels of β-carotene and vitamin A in serums were significantly decreased compared to control [290]. Vitamin A deficiency promoted Aβ accumulation [291] and the rate of cognitive decline negatively correlated with serum level of vitamin A in the elderly [292]. Transgenic AD mice treated with vitamin A intraperitoneally for 8 weeks showed decreased cerebral tau phosphorylation and Aβ deposition, decreased microglia and astrocyte activation, attenuated neurodegeneration and improved spatial memory and learning [293]. Despite some promising results in animal models, there has been a lack of human clinical trials for vitamin A or carotenoids in the AD treatment. 

### 18.2. Vitamin D

Vitamin D belongs to the secosteroids under the category of sterol but is placed with two others vitamins categorized as prenol lipids here for the convenience of discussion. Human studies revealed a correlation between low circulating 25-hydroxyvitamin D (25-OHD) level and dementia [294]. In AD hippocampal CA1 cells, vitamin D hormone receptor (VDR) mRNA is downregulated [295]. VDR has a role in reducing cerebral soluble and insoluble Aβ [296]. In AD rat model, vitamin D3 enriched diet led to decreased Aβ peptides and amyloid plaques, reduced inflammation and increased nerve growth factors in the brains. Vitamin D also modulated age-related increase in proinflammatory state. As a result, vitamin D supplement enhanced the performance of learning and memory both in aging and AD models [297].

### 18.3. Vitamin E

Vitamin E is composed of four tocopherols and four tocotrienols with antioxidant properties. Among all vitamin E isomers, RRR-*α*-tocopherol has the highest in vivo bioactivity and is the only isoform that is essential for humans [298]. Vitamin E is rich in unsaturated FAs such as DHA and AA and has high concentrations in the brain and retina. It is located within cell membranes, which has functions in stabilizing the membrane, protecting DHA from oxidative damage, and promoting membrane repair [298,299]. A meta-analysis conducted in 2018 found that vitamin E levels are lower in AD patients compared to cognitively normal subjects [300], although a few case-control and genome-wide association studies found no association [301,302]. Animal studies showed positive effects of vitamin E supplement in mitigating cognitive decline by reducing Aβ load [303]. Vitamin E treatment also slows the progression of the disease in patients with moderately severe AD. However, a Cochrane review in 2017 including four double-blind, randomized trials and another one trial published in the same year found no evidence that vitamin E prevents progression to dementia nor did it improve cognition in MCI, dementia or AD patients [304,305], except one study conducted by Dysken et al., which showed slower functional decline, with a delay in clinical progression of 19% per year in the vitamin E-treated group [306]. One recent study pointed out factors that may affect the bioavailability and effectiveness of vitamin E supplements, such as the patients’ baseline vitamin E level, the isoform or the source of vitamin E used for treatment, and genetic variants [307,308].

## 19. Lipidomic Studies

In lipidomic analyses, AD patients are characterized by diminished ether sterols, PCs, SMs and phospholipids compared to healthy control [309]. According to Kim et al., there were 14 significantly elevated lipids in the plasma of AD, exhibiting >2-fold increases in LDL/VLDL including PE, DAG, TG and ceramide. Three lipid species (TG 50:1, DAG 18:1_18:1, and PE 36:2) showed a high correlation with the degree of brain atrophy and could be utilized as candidates in differentiating the early stage of MCI when used with MMSE [275]. Using a LC/MS-based nontargeted metabolomics approach, Trushina et al. found that sphingolipids and cholesterol transport were altered in both AD patient’s plasma and CSF when compared to cognitively normal individuals [310].

## 20. Conclusions

**Figure 1:**
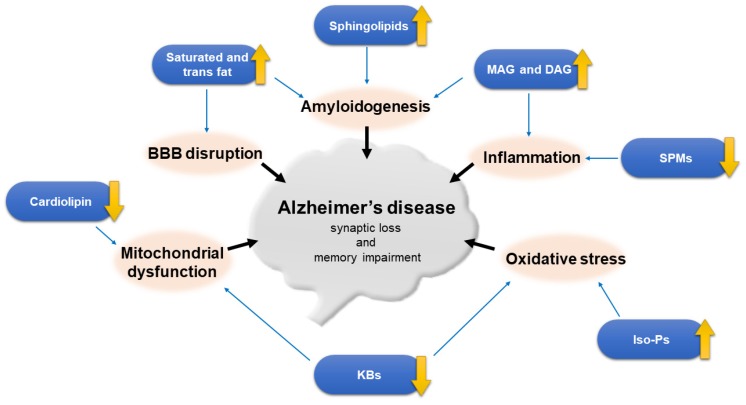
Roles of lipids in AD pathogenesis.

Dysregulated lipid homeostasis is associated with aging and contributes greatly to the pathogenesis of AD. The mechanisms linking lipid dysregulation and AD consist of alterations in intestinal microbiota and the gut-brain axis, neuronal signaling pathway, BBB disruption, mitochondrial dysfunction, oxidative stress, and inflammation, which together lead to synaptic loss and ultimately memory impairment. Some lipids play essential roles in AD pathogenesis and have been proposed as biomarkers (Figure 1). Disturbances of lipids in AD brains fell into the following categories:

1. Saturated and trans fats would result in BBB dysfunction and Aβ aggregation, and were strongly associated with risk of AD, thus should be eliminated from diets and substituted with unsaturated fats if possible.
2. Among PUFAs, n-3 and n-9 FAs are beneficial, while n-6 FAs such as arachidonic acid (AA) are harmful to the brain in terms of cognitive function. DHA level is decreased in AD brains, and its supplementation is therapeutically promising especially when given early, however some randomized controlled trials showed no cognitive benefits. The lipid mediators of PUFAs called specialized pro-resolving mediators (SPMs) play a crucial role in the anti-inflammatory response and are decreased in AD brains. Isoprostanes such as F_2_-isoprostanes ( F2- IsoPs) and Neuro-Ps are increased as a consequence of increased oxidative stress and lipid peroxidation in AD patients.
3. Elevated glycerolipids such as triglyceride (TG) and diacylglycerol (DAG) have been reported to correlate with AD but their roles are less well defined. Monoacylglycerol lipase (MAGL) inhibitor seems to hold potential for ameliorating AD pathology.
4. Glycerophospholipids are the main lipid components in the cell membranes. Ethanolamine plasmalogens are reduced early in the AD brains. The change in choline plasmalogens are less consistent but phospholipid deacylation products, e.g., glycerophosphocholine are increased. Phospholipase A2 (PLA2) and D1 (PLD1) are increased in AD.
5. Among the sphingolipids, increased levels of sphingomyelin and ceramide are found in AD brains, although a few studies showed a decrease or no change in sphingomyelin. Sphingomyelinase—particularly acid and neural sphingomyelinase 2—are upregulated in AD. Sphingosine 1-phosphate (S1P) and sulfatide are depleted from the earliest stages of AD. Ganglio-series gangliosides are decreased while simple gangliosides such as GM2, GM3, GD3 and GM4 are elevated in AD brains.
6. The influences of cholesterol on AD are controversial among epidemiologic and lipidomic studies both in animals and humans. It seems that increased levels of mid-life plasma cholesterol are associated with elevated AD risk and use of statins seems beneficial when introduced at this age, whereas late-life cholesterol levels are not associated with AD risk; statin treatment at this age also has no impact on risk of dementia. Oxysterols such as 27-hydroxycholesterol (27-OHC) and 24S-hydroxycholesterol (24S-OHC) are elevated in the CSF and brain of AD patients, but level of 24S-OHC is decreased late in the disease course. Therefore, brain cholesterol homeostasis should be regulated within an appropriate range and neither low nor high levels are healthy at all. The ApoE4 allele carrying status portends a decreased age of onset and an increased risk in AD through an allele-dependent manner. ABC transporters are associated with cholesterol transport and play important roles in Aβ efflux and amyloidogenesis.
7. Fat-soluble vitamins A, D and E are regarded as anti-oxidants with potential benefits and the serum levels of these vitamins are decreased in AD patients compared to cognitively normal individuals. However, randomized-controlled trials fail to support their routine use, except one study which showed slower cognitive decline in the vitamin E-treated group.
8. A Toll-like receptor 4 agonist, monophosphoryl lipid A (MPL), can stimulate uptake of Aβ by microglia and has been investigated for its treatment application.
9. Ketogenic diets seem to hold potential in ameliorating cognitive decline during treatment, however, whether this unnatural diet is suitable for long-term usage needs further investigation.

Researches have shown that the effects of FAs on AD diagnosis and progression are not straightforward, and multiple lipids need to be accounted for to gain an accurate insight. In an era with rising AD prevalence when the ideal treatment is yet to be found, lifestyle modifications such as changing dietary lipid content seem to be a practical and natural way of facing the disease. However, since humans are omnivores, consuming lipids as well as other energy sources and trace elements, studies of dietary effects on AD are difficult and diet modification is far from ideal. Nevertheless, due to the abundance of lipids in the brain, knowing the effects of lipids on the pathogenesis of AD is valuable for disease modification. We are anticipating that the continuous exploration of the metabolome including the lipidome leads to further progression in this field.

## Figures and Tables

**Table 1:** Lipid categories.

Category	Example
Fatty acids (FA)	arachidonic acid (AA), docosahexaenoic acid (DHA)
Glycerolipids (GL)	monoacylglycerol (MAG), diacylglycerol (DAG), triacylglycerol (also triglyceride, TG)
Glycerophospholipids (GP)	phosphatidylethanolamine (PE), phosphatidylcholine (PC), phosphatidylserine (PS)
Sphingolipids (SP)	sphingomyelin, ceramide, sulfatide, ganglioside
Sterol lipids (SL)	cholesterol, vitamin D
Prenol lipids (PR)	carotenoid, vitamin E & K
Saccharolipids (SL)	lipid A
Polyketides (PK)	lovastatin

## Abbreviations

AD	Alzheimer disease
Aβ	β-amyloid
NFTs	neurofibrillary tangles
LC-PUFAs	Long-chain polyunsaturated fatty acids
FAs	fatty acids
DHA	docosahexaenoic acid
AA	arachidonic acid
ApoE	apolipoprotein E
CLU	clusterin
SORL1	sortilin-related receptor 1
ABCA 7	ATP-binding cassette, sub-family A, member 7
GL	Glycerolipids
MAG	monoacylglycerol
DAG	diacylglycerol
TG	triacylglycerol
GP	Glycerophospholipids
PE	phosphatidylethanolamine
PC	phosphatidylcholine
PS	phosphatidylserine
SP	Sphingolipids
SL	Sterol lipids
PR	Prenol lipids
SL	Saccharolipids
PK	Polyketides
APP	amyloid protein precursor
SFA	saturated fatty acids
MUFAs	monounsaturated fatty acids
SPMs	specialized proresolving lipid mediators
SCFAs	short-chain FAs
LA	linoleic acid
ALA	α-linolenic acid
EPA	eicosapentaenoic acid
OL	oleic acid
SA	stearic acid
THA	tetracosahexaenoic acid
PS1	presenilin 1
c-JNK	c-Jun N-terminal kinases
LO	lipoxygenase
COX	cyclooxygenase
PEP	prolyl endopeptidase
IsoPs	Isoprostanes
NeuroPs	Neuroprostanes
KD	Ketogenic diet
KBs	ketone bodies
MCT1	monocarboxylic acid transporter 1
NMDA	N-methyl-d-aspartate
PlsEtns	ethanolamine plasmalogen
PI	Phosphoinositide
PLD	Phospholipase D
SMs	sphingomyelins
S1P	sphingosine 1-phosphate
BACE1	β-secretase 1
RxRγ	retinoid X receptor gamma
MPL	Monophosphoryl lipid A
LDL	low-density lipoprotein
HDL	high-density lipoprotein
